# Management of sugar dust in the sugar industry

**DOI:** 10.1016/j.heliyon.2023.e23158

**Published:** 2023-12-05

**Authors:** Kingsley O. Iwuozor, Toluwalase Ojeyemi, Ebuka Chizitere Emenike, Chisom T. Umeh, Abel Egbemhenghe, Bridget Dunoi Ayoku, Tolu I. Ogunsanya, Samuel Ogunniyi, Joshua O. Ighalo, Adewale George Adeniyi

**Affiliations:** a Nigeria Sugar Institute, Ilorin, Nigeria; b Department of Pure and Industrial Chemistry, Nnamdi Azikiwe University, P. M. B. 5025, Awka, Nigeria; c Department of Environmental Toxicology, Texas Tech University, USA; d Department of Crop Protection and Environmental Biology, University of Ibadan, Nigeria; e Department of Chemistry and Biochemistry, College of Art and Science, Texas Tech University, USA; f Department of Chemistry, Lagos State University, Ojo, Lagos, Nigeria; g Department of Pure and Industrial Chemistry, University of Port Harcourt, Rivers state, Nigeria; h Department of Chemistry, University of Lagos, Lagos state, Nigeria; i Department of Chemical Engineering, University of Ilorin, P. M. B. 1515, Ilorin, Nigeria; j Department of Chemical Engineering, Nnamdi Azikiwe University, P. M. B. 5025, Awka, Nigeria; k Department of Chemical Engineering, Kansas State University, Manhattan, KS, USA; l Chemical Engineering Department, Landmark University, Omu-Aran, Nigeria

**Keywords:** Dust monitoring, Industrial dust, Nanotechnology, Ventilation systems, Sugar industry safety

## Abstract

Sugar dust poses significant risks in the sugar industry, threatening workers' safety and health as well as the potential for explosions and fires. The combustibility of sugar dust arises from its small, lightweight particles that disperse easily and ignite readily. Effective management strategies are essential to ensuring a safe work environment and preventing accidents. This perspective article provides an overview of sugar dust management in the global sugar industry. Various methods are employed to collect and manage sugar dust, including dust collectors, air handling systems, and proper housekeeping procedures. Advancements like electrostatic precipitators, high-efficiency particulate air filters, and self-cleaning dust collection systems show promise for future management. Utilizing both artificial intelligence and nanotechnology can also contribute to minimizing the concentrations of sugar dust in facilities. Stringent regulations and guidelines exist to control dust explosions in the industry. Implementation of robust safety measures and training programs significantly curbs the economic and environmental toll of sugar dust explosions. The paper concludes with recommendations to address sugar dust challenges, including enhanced regulation, investment in technology and research, and improved collaboration among industry stakeholders. These measures will mitigate hazards, ensure worker well-being, and safeguard the sugar industry's operations.

## 1 Introduction

Sugar is one of the most widely produced and traded commodities in the world, with global production reaching approximately 169 million metric tons in 2020 [[1], [2], [3]]. The sugar industry involves several stages, from growing and harvesting sugar cane or sugar beet, to processing and refining the raw materials into various sugar products, and finally transporting the sugar products to markets around the world [4,5]. Sugar production typically takes place in various regions, with Brazil, India, and Thailand being among the top producers of sugar cane and the European Union and Russia being among the top producers of sugar beet [6,7]. After harvesting, the raw sugar cane or beet is transported to processing facilities, where it undergoes several stages of refining to remove impurities and produce various sugar products, including granulated sugar, powdered sugar, and molasses [8,9]. Once the sugar products are refined, they are typically transported via ship, truck, or rail to various markets around the world.

Sugar dust is a fine powder that is produced when sugar is processed and handled. It is a by-product of the refining processes that are used to produce granulated sugar from sugar cane or sugar beet [10]. Sugar dust is composed of tiny sugar particles that are released into the air as sugar is ground, dried, and transported. The prevalence of sugar dust in the sugar industry is significant. The dust can be generated by the mechanical handling of the sugar, such as during the grinding, screening, and sieving of the sugar crystals [11]. It could also be generated by the movement of bulk sugar in storage and transportation, such as during the loading or unloading of sugar from trucks, ships, or storage silos. The high level of sugar dust in the sugar industry poses a significant risk to workers and equipment. The fine particles of sugar can be highly combustible, and the buildup of sugar dust can lead to incidents of sugar dust explosions. Sugar dust explosions can be highly destructive, leading to loss of life, serious injury, and significant damage to equipment and facilities [12]. In addition to the risk of explosion, sugar dust can also pose health risks to workers in the sugar industry. Inhaling sugar dust may result in respiratory issues like asthma., and exposure to sugar dust can also lead to skin irritation and eye irritation [13].

There have been several incidents of explosions caused by sugar dust within the sugar industry in recent years. For instance, in September 2007, a sugar refinery belonging to the Prodimex Group in Russia experienced a blast that resulted in the deaths of three individuals and injuries to several others [14]. A year later, in February 2008, in Port Wentworth, Georgia, USA, the Imperial Sugar Plant witnessed a substantial dust explosion in which 14 individuals lost their lives, and numerous others sustained injuries. The buildup of sugar dust on surfaces and equipment, which became airborne and ignited led to the occurrence of the explosion [15]. In November 2007, an explosion occurred at a sugar silo in the Domino Sugar Refinery in Baltimore, USA, which fortunately did not result in any reported deaths. The explosion was due to the accumulation of sugar dust in the refinery [16].

In October 2021, at least 41,000 tons of sugar were destroyed in a fire that broke out at the Komati Sugar Mill in the Mpumalanga province of South Africa. The presence of sugar dust on a conveyor belt fueled the inferno [17]. A similar fire was reported at Wilmar Sugar Limited's Pioneer Mill in Queensland, Australia, in October 2019 [18]. A month later, still in Queensland, Mackay Sugar Limited reported that the sugar dust fueled a fire caused by the high temperatures of the conveyor belt [19]. In November 2021, a fire occurred on a conveyor belt carrying sugarcane bagasse at a sugar and ethanol production facility owned by Usina Santa Lucia in Sao Paulo, Brazil, but the presence of sugar dust further fueled the flames [20]. The risks associated with sugar dust are not ones that should be overlooked, especially by the quality control and assurance personnel in the factory, and their prevalence has necessitated the need for this perspective article.

This paper is aimed at discussing the presence of sugar dust in the industry, discussing measures currently put in place to manage it, and suggesting measures that could be further taken to ensure its further concentration reduction in sugar production facilities. A deep-seated commitment to enhancing safety, environmental sustainability, regulatory compliance, and operational efficiency in the sugar production process are the primary motivators for this study. By providing a better understanding of sugar dust and evaluating effective mitigation strategies, the authors intend to offer valuable insights to industry professionals, policymakers, and researchers. The compelling rationale for this study lies in the imperative to mitigate safety risks, minimize environmental impact, meet evolving regulatory standards, and optimize industrial practices. It is important to clarify that this study does not present original research findings or new statistical analyses, but rather synthesizes and interprets existing knowledge and industry best practices with a view to discussing better techniques for the management of sugar dust.

## 2 Negative impacts of sugar dust

Sugar dust, like many other types of combustible dust, has the potential to ignite and cause explosions. When sugar dust particles become airborne, they can create a combustible mixture with air. If an ignition source is introduced, such as a spark, the mixture can ignite and cause an explosion. This phenomenon is known as a dust explosion, and it can be incredibly dangerous [21]. The potential for dust explosions within the sugar industry is particularly high due to the large volumes of sugar that are handled and processed in sugar mills and refineries. Sugar dust can accumulate on surfaces and equipment, creating a hazard if it becomes airborne [22]. In addition, the handling of sugar can generate a lot of dust, especially during conveying and packaging operations. Sugar dust explosions can cause significant damage to equipment and facilities, as well as pose a serious risk to workers' safety. In addition, the resulting fires can be difficult to extinguish and can spread quickly through a facility [22].

The sugar industry faces a notable danger from secondary explosions, intensifying the risks linked with sugar dust. These explosions can lead to catastrophic losses, emphasizing the need for a comprehensive understanding of their causes, consequences, and preventive measures. Primary explosions often trigger secondary explosions, dispersing accumulated sugar dust into the surrounding environment [12]. Factors such as ignition sources, disrupted equipment, or further dust accumulation can act as triggers for secondary explosions. When these factors converge, the potential for a devastating chain reaction increases [12]. Secondary explosions in the sugar industry can have dire consequences. These explosions can unleash destructive forces, causing extensive damage to facilities, equipment, and, most importantly, human lives. Past incidents serve as reminders of the urgency to address the risks associated with secondary explosions promptly.

Exposure to sugar dust can pose significant health risks to workers in the sugar industry. Breathing in sugar dust may result in respiratory issues, such as coughing, wheezing, and difficulty in breathing. In addition, prolonged exposure to sugar dust can cause chronic bronchitis, emphysema, and even lung cancer [23]. Workers in the sugar industry are particularly at risk of exposure to sugar dust, as they may be involved in tasks such as loading and unloading sugar, handling bags of sugar, or operating machinery that generates dust. Sugar dust explosions can have severe economic and environmental impacts. The financial costs associated with these explosions can be extensive, ranging from property damage, equipment replacement, and lawsuits to worker compensation and increased insurance premiums [16]. These costs can be especially devastating for small businesses and companies in developing countries where safety regulations may be lax or poorly enforced. Additionally, explosions caused by sugar dust can result in significant environmental ramifications, including the pollution of both air and water. The clean-up and disposal of debris from an explosion can lead to increased emissions of particulate matter, potentially causing health problems for nearby communities. Furthermore, the use of fire-fighting chemicals and water to contain the explosion can have harmful effects on the environment, particularly on nearby water sources [24]. These environmental impacts can lead to long-term ecological damage, including the destruction of habitats and the loss of biodiversity. In addition to the direct costs of sugar dust explosions, there are also indirect costs to consider. These include the loss of productivity and revenue due to the shutdown of production facilities, the negative impact on a company's reputation, and the potential for decreased investor confidence.

## 3 Recent initiatives in the sugar industry to manage sugar dust

Based on the impacts of sugar dust, it is crucial to have effective methods for collecting and managing sugar dust to minimize its negative impacts. There are several methods for collecting and managing sugar dust in the sugar industry. One of the most common methods is the use of dust collectors, which are engineered for the capture and containment of dust particles generated during various stages of the sugar production process [25]. Dust collectors are specialized pieces of equipment engineered for the capture and elimination of airborne particles, including sugar dust, from industrial processes. There are several types of dust collectors used in the sugar industry, including baghouse filters, cyclone separators, and electrostatic precipitators [26]. Baghouse filters are the most commonly used dust collectors in the sugar industry. They work by trapping dust particles on the surface of filter bags, which are periodically cleaned to remove the accumulated dust [27]. Cyclone separators use centrifugal force to separate dust particles from the air, while electrostatic precipitators utilize an electric charge to draw in and gather particles of dust [28]. Dust collectors are strategically installed at various points in the sugar processing and handling systems, including conveyor transfer points, sugar silos, packaging machines, and within equipment. These dust collectors serve a dual purpose: capturing fugitive dust that may have escaped equipment and removing internal dust buildup. By effectively capturing sugar dust before it can accumulate in the air, dust collectors play a vital role in safeguarding workers safety and averting the danger of explosions in the sugar industry [28].

Another effective method for managing sugar dust is the use of air handling systems, which are designed to circulate air in the sugar production area and remove dust particles. While air handling systems can play a role in maintaining clean and safe air in the sugar production area, it is important to clarify their purpose and limitations. Air handling systems, including room ventilation systems, should primarily be used to handle incidental amounts of dust that escape local dust collection systems. They are not intended to remove significant amounts of dust from production facilities. Instead, their primary function is to circulate air and provide a comfortable working environment. It is essential to adhere to industry standards, such as NFPA 654, which emphasize the importance of local dust collection systems for effective dust management [29]. However, it is still appropriate to incorporate high-efficiency particulate air (HEPA) filters on the ventilation system exhaust intakes as an additional measure to enhance air quality [30].

In addition to these methods, implementing effective housekeeping procedures is crucial for the management of sugar dust. These procedures may involve regular cleaning of equipment and production areas. It is important to note that when using vacuum cleaning systems, particularly portable vacuum cleaners, specific safety considerations must be taken into account. They should be appropriately rated for use with combustible dust to prevent them from becoming an ignition source. By selecting vacuum cleaners designed for handling combustible dust, the risk of potential ignition can be minimized, ensuring the safety of workers and the facility [22].

The potential for sugar dust explosions is one such risk that has been addressed through various regulations and standards across different countries. Governments around the world have enacted regulations to prevent such explosions and ensure the safe handling and storage of sugar and other combustible dusts [11]. In the European Union, the European Agency for Safety and Health at Work has developed guidelines for preventing dust explosions in the workplace, including requirements for hazard assessments, risk management plans, and employee training [31]. These guidelines recommend measures such as proper ventilation, cleaning, and the use of explosion-proof equipment to minimize the risk of explosions. In addition, the International Electrotechnical Commission (IEC) has developed standards for equipment used in potentially explosive atmospheres, including those that may arise from sugar dust [32].

It is essential to adhere to the combustible dust safety standards established by the National Fire Protection Association (NFPA) to ensure the highest level of safety in sugar industry facilities. The relevant NFPA standards for sugar industry operations include NFPA 61, NFPA 652, and NFPA 654. NFPA 61 offers recommendations to prevent fires and explosions in facilities engaged in agricultural and food processing, which are applicable to sugar production. NFPA 652 outlines fundamental principles and criteria for recognizing and controlling the fire and explosion hazards associated with combustible dusts. Furthermore, NFPA 654 addresses the guidelines for averting fire and dust explosions in the manufacturing, processing, and handling of combustible particulate solids, such as sugar. Adhering to these standards is crucial for establishing safe practices, managing risks associated with sugar dust, and ensuring the well-being of workers and the integrity of the facility [29,33]. In addition to these regulatory requirements, many companies in the sugar industry have implemented their own dust management systems and procedures. These may include the use of dust collection systems, the implementation of cleaning schedules, and the regular monitoring of dust levels in the workplace.

## 4 Emerging technologies and additional measures

**Fig. 1:**
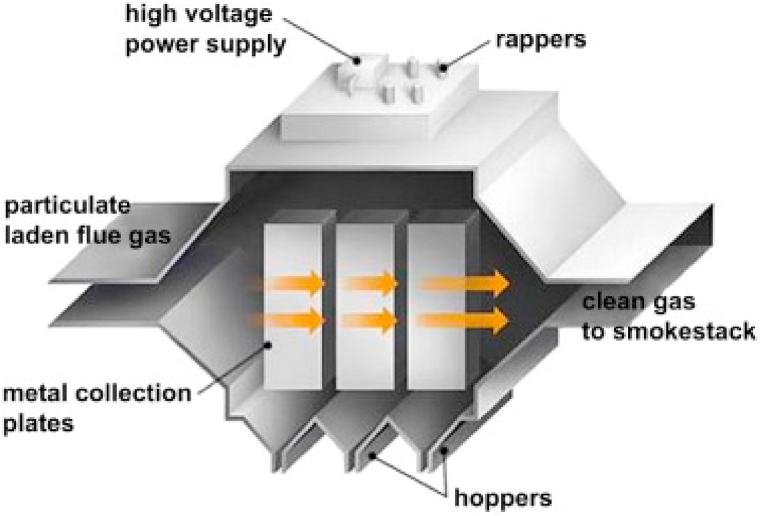
Schematic of Electrostatic Precipitator. *Reproduced from Cheremisinoff, Rosenfeld* [43] *with permission from Elsevier.*

Sugar dust collection technology has advanced significantly over the years with the introduction of innovative solutions that address the challenges posed by sugar dust in the sugar industry. The use of advanced technologies for sugar dust collection has the potential to improve the safety and efficiency of sugar production, minimize the likelihood of dust explosions, and minimize the environmental impact of sugar dust. One example of an innovative solution for sugar dust collection is the use of electrostatic precipitators (ESPs). An ESP (Fig. 1) is an air pollution control device that efficiently removes fine particulate matter, such as dust and smoke, from industrial emissions and air purification systems [34]. It operates on the principle of electrostatic attraction, using a high-voltage electrical field to charge airborne particles as they pass through. These charged particles are then attracted to oppositely charged collection plates or electrodes within the ESP [35]. This process effectively captures and removes the particles from the air stream, improving air quality and reducing pollution. ESP components typically include charging electrodes, collection plates, and a power supply. The charging electrodes apply a strong electrical charge to the particles, causing them to become electrically charged [36]. As the charged particles move toward the collection plates, they are attracted to and adhere to the plates' surfaces. This technology has been proven effective in capturing sugar dust at various stages of the sugar production process, including during milling, conveying, and packaging [37,38].

Another emerging technology in the sugar industry is the use of high-efficiency particulate air (HEPA) filters. HEPA filters excel at effectively trapping minute particles, such as dust, pollen, pet dander, smoke, and even some bacteria and viruses from the air due to their high efficiency in filtration. These filters are commonly used in air purifiers, vacuum cleaners, and ventilation systems to improve indoor air quality [39,40]. Additionally, advancements in automation technology have allowed for the development of self-cleaning dust collection systems. These systems have the capability to autonomously identify and eliminate dust from production machinery, decreasing the necessity for manual cleaning and mitigating the potential for dust buildup [41]. Certain systems go a step further by employing artificial intelligence to enhance the optimization of collection and cleaning schedules, thereby increasing efficiency and cutting down on costs [42]. Furthermore, there has been a shift towards developing sustainable solutions for sugar dust collection.

The application of robotics and automation stands out as a promising technology poised to transform the management of sugar dust. Utilizing robotic systems enables the automatic cleaning and upkeep of production equipment, diminishing the reliance on manual cleaning and lowering the likelihood of dust accumulation [44]. Furthermore, automation technology proves valuable in optimizing schedules for dust collection and cleaning, thereby enhancing efficiency and cutting down on costs. Another inventive approach involves the incorporation of nanotechnology in sugar dust management. Nanoparticles, characterized by a particle size ranging from 1 to 100 nm and composed of natural or artificial polymers, offer a solution [[45], [46], [47], [48], [49], [50]]. These nanoparticles exhibit effectiveness in capturing and eliminating sugar dust particles, ultimately enhancing air quality and mitigating the risk of dust explosions. Moreover, nanotechnology can be harnessed to design more resourceful and environmentally sustainable sugar dust collection systems, thereby lessening the ecological impact associated with sugar production [51].

Governments, industry groups, and individual companies must collaborate to ensure they take all necessary measures to prevent these types of accidents and protect workers and the environment from the potential harm caused by sugar dust explosions. Individual companies or factories should ensure regular cleaning of equipment and surfaces, the use of explosion-proof equipment, and the implementation of dust control measures. To protect workers from the health risks associated with sugar dust exposure, it is important for employers in the sugar industry to implement appropriate safety measures. These may include providing workers with personal protective equipment, such as respirators, and flame-resistant clothing, and ensuring that machinery is properly maintained to minimize dust generation. In addition, regular monitoring of air quality in the workplace can help identify areas where sugar dust levels may be particularly high, and measures can be taken to address these issues. The respective factories can also promote worker safety by providing education and training programs. The design of these initiatives should aim to provide education to the workforce on the dangers of sugar dust explosions and the steps they can take to prevent them. They should also provide training on how to properly use equipment and handle sugar to minimize the risk of explosions.

The resulting explosion from sugar dust can be devastating, causing injury or death to workers and damaging equipment and structures. As a result, it is essential that the government make some contribution to preventing sugar dust explosions and promoting worker safety. One way that the government can prevent sugar dust explosions is by implementing regulations and standards for sugar processing facilities. These regulations should require facilities to regularly inspect and maintain their equipment to prevent the accumulation of sugar dust. They should also require facilities to install ventilation systems to keep the air clear of sugar dust and to use explosion-proof electrical equipment to prevent sparks and other ignition sources. Enforcement mechanisms are essential to ensuring compliance with regulations and standards. Government agencies should establish monitoring systems to assess sugar processing facilities regularly, ensuring their adherence to safety guidelines. Penalties, such as fines and temporary shutdowns, may be imposed on non-compliant facilities to promote a culture of safety and encourage compliance.

Finally, it is crucial for government entities at various levels to play their respective roles in responding to sugar dust explosions. Local governments should take the lead in providing immediate emergency response services to swiftly address an explosion and mitigate its impact on the local community and facilities. This includes coordinating with local fire departments, emergency medical services, and relevant authorities to ensure a prompt and effective response. At the national and/or regional level, the government's role becomes more focused on broader actions following a sugar dust explosion. This encompasses conducting thorough investigations to determine the root causes of the incident and implementing corrective measures. The national or regional government can collaborate with industry experts, regulatory bodies, and stakeholders to establish updated safety standards, guidelines, and best practices for the sugar industry. These measures can help enhance overall safety and prevent future occurrences of sugar dust explosions.

Quantitative risk assessment methods hold significant potential for enhancing the industry's safety and risk management practices. Future research can delve deeper into these quantitative approaches, offering a more detailed examination of their applicability and benefits within the sugar industry. Such investigations can further enrich the technical depth of sugar dust management strategies and contribute to the holistic improvement of safety practices in this vital sector. While insights into the evolving landscape of sugar dust management and its potential future directions have been provided, it is important to recognize the importance of addressing practical implementation aspects. Organizations may encounter a variety of challenges when adopting advanced sugar dust management technologies and strategies. These challenges can range from initial capital investment to adapting existing infrastructure and processes. However, it's essential to highlight that overcoming these challenges is crucial for improving worker safety, environmental sustainability, and operational efficiency in the sugar industry. Potential solutions can include phased implementation, collaboration with technology providers, and leveraging government incentives for adopting innovative technologies. By carefully considering these challenges and solutions, sugar producers can navigate the path toward more effective and sustainable sugar dust management.

Quantitative Risk Assessment (QRA) in sugar industries involves a structured analysis of potential dust explosion risks, using various methods and approaches to assess both the probability and severity of such events. For probability analysis, one commonly used method is Fault Tree Analysis (FTA). FTA breaks down the potential causes of a dust explosion into logical, interconnected events and quantifies the likelihood of each event occurring. For instance, it may examine factors like dust concentration, ignition sources, and containment measures. Similarly, Event Tree Analysis (ETA) assesses the sequence of events following a dust explosion, evaluating the consequences and their probabilities. In terms of severity analysis, one approach is Consequence Analysis, which examines the potential impact of a dust explosion. This involves evaluating factors such as the explosion overpressure, thermal radiation, and toxic gas release. These consequences can be quantified using computational models like Computational Fluid Dynamics (CFD) simulations and dispersion models. By combining the results of probability and severity analyses, QRA provides a comprehensive assessment of the overall risk associated with dust explosions in sugar industries. This information is invaluable for decision-makers to prioritize safety measures, allocate resources effectively, and ensure that the appropriate control measures are in place to mitigate the hazards, ultimately safeguarding the well-being of workers, protecting assets, and preventing potentially devastating incidents.

## 5 Conclusion

In conclusion, sugar dust is a major concern in the sugar industry due to its potential hazards to workers' health and safety. Therefore, effective management strategies for sugar dust are crucial to maintaining a safe working environment and preventing accidents. Based on this study, several key measures that have been utilized to help mitigate sugar dust risks in the sugar industry have been identified. These include regular cleaning of equipment and work areas, using appropriate ventilation and dust collection systems, implementing effective housekeeping practices, and providing appropriate personal protective equipment (PPE) to workers. To effectively address sugar dust in the sugar industry, there needs to be greater collaboration and communication among industry stakeholders, including government regulators, industry groups, and individual companies. This can include the development of best practices and guidelines for preventing and mitigating sugar dust, as well as the establishment of consistent safety protocols and regulations across the industry. In addition, there needs to be greater investment in research and development to develop new technologies and equipment to prevent sugar dust from accumulating and spreading in sugar production facilities. This can include the development of new ventilation systems, explosion-proof equipment, and other tools and technologies to improve safety. Ultimately, addressing the challenges posed by sugar dust requires a concerted effort from all stakeholders in the sugar supply chain. By working together to develop new safety protocols, invest in research and development, and prioritize worker safety, we can ensure a safer and more sustainable future for the global sugar supply chain.

## Funding

There was no external funding for the study.

## Compliance with ethical standards

This article does not contain any studies involving human or animal subjects.

## Data availability statement

No data was used for the research described in the article.

## CRediT authorship contribution statement

**Kingsley O. Iwuozor:** Writing - review & editing, Writing - original draft, Visualization, Supervision, Resources, Methodology, Investigation, Conceptualization. **Toluwalase Ojeyemi:** Writing - review & editing, Writing - original draft, Visualization, Methodology, Investigation. **Ebuka Chizitere Emenike:** Writing - review & editing, Writing - original draft, Visualization, Resources, Methodology, Investigation. **Chisom T. Umeh:** Writing - review & editing, Writing - original draft, Visualization, Methodology, Investigation. **Abel Egbemhenghe:** Writing - review & editing, Writing - original draft, Visualization, Methodology, Investigation. **Bridget Dunoi Ayoku:** Writing - review & editing, Writing - original draft, Visualization, Methodology, Investigation. **Tolu I. Ogunsanya:** Writing - review & editing, Writing - original draft, Visualization, Methodology, Investigation. **Samuel Ogunniyi:** Writing - review & editing, Writing - original draft, Visualization, Methodology, Investigation. **Joshua O. Ighalo:** Writing - review & editing, Writing - original draft, Visualization, Methodology, Investigation. **Adewale George Adeniyi:** Writing - review & editing, Writing - original draft, Visualization, Supervision, Resources, Methodology, Investigation.

## Declaration of competing interest

The authors declare that they have no known competing financial interests or personal relationships that could have appeared to influence the work reported in this paper.
